# Experimental and DEM Study on the Mechanical Behaviors of Sand–Fines Mixtures with Different Fines Contents and Particle Size Ratios

**DOI:** 10.3390/ma18214929

**Published:** 2025-10-28

**Authors:** Kejia Wu, Bing Lv, Hexige Baoyin, Dongsheng Li, Zhouyi Yan, Pengqiang Yu, Yang Liu

**Affiliations:** 1 Department of Civil Engineering, University of Science and Technology Beijing, Beijing 100083, China; d202110020@xs.ustb.edu.cn (K.W.); h_baoyin@xs.ustb.edu.cn (H.B.); m202320165@xs.ustb.edu.cn (D.L.); yanzhouyi1996@126.com (Z.Y.); pq_yu@ustb.edu.cn (P.Y.); 2 Second Engineering of CCCC Third Highway Engineering Co., Ltd., Tianjin 301800, China; 18901197107@189.cn; 3 School of Civil and Architectural Engineering, Liaoning University of Technology, Jinzhou 121001, China

**Keywords:** sand–fines mixtures, fines content, particle size ratio, triaxial tests, discrete element method, shear strength, fabric anisotropy

## Abstract

To clarify the regulatory laws of the fines content (*FC*) and particle size ratio (*SR*) on the mechanical properties of sand–fines mixtures and reveal the underlying microscopic mechanical mechanisms, this study takes sand–fines mixtures composed of natural river sand and silt as the research object. It systematically investigates the macro-mechanical behaviors and micro-interaction mechanisms of the mixtures by combining laboratory triaxial tests and discrete element method (*DEM*) simulations. First, through conducting triaxial drained shear tests on mixtures with three particle size ratios (*SR* = 9.1, 18.7, and 39.7) under seven fines contents (*FC* = 0%, 10%, 20%, 30%, 50%, 70%, and 100%), it is found that both the peak friction angle (*φ_ps_*) and critical-state friction angle (*φ_cs_*) of sand–fines mixtures show a “first increase, then decrease” trend with the increase in *FC*. The peak inflection points of their variation curves are the threshold fines content related to *SR*; meanwhile, a fines content below this threshold has an inhibitory effect on dilatancy, while that above this threshold exerts a promotive effect on dilatancy. Subsequently, by exploring the microscopic contact behaviors of sand–fines mixtures, it is observed that, under the fines content corresponding to the highest peak strength, the strong contact network and weak contact network inside the material form an optimal coordination between efficient load-bearing and stable support. This coordination enables the macro-strength of the mixture to reach the peak at this fines content. In addition, by modifying the weight coefficient of fabric anisotropy, a unique linear relationship between the fabric anisotropy of strong contacts and the stress ratio can be established, confirming that the strong contact network plays a core mechanical role in mixtures with different *FC* values.

## 1. Introduction

Binary mixtures, typically including gravel–sand mixtures and sand–silt mixtures [1], are widely used in roadbed fill, the seepage-proof body of dikes and dams, tailings dam accumulations, and the foundations of marine engineering structures (e.g., wharf core filling, and wind power foundation cushion) [2,3,4]. Their strength indicators (such as the peak friction angle and critical-state friction angle) and deformation characteristics (such as the dilatancy effect) directly determine the short-term anti-sliding stability and long-term deformation performance of engineering structures. As key material parameters for regulating binary mixtures, the fines content (*FC*) [5,6,7] and particle size ratio (*SR*) [8,9] significantly affect the strength and deformation characteristics of the mixtures.

At the current stage, scholars have conducted systematic studies on the strength indicators (peak strength friction angle *φ_ps_* and critical-state friction angle *φ_cs_*) of binary mixed materials through laboratory triaxial tests. In the research on peak strength (*φ_ps_*), there are clear disagreements among scholars regarding the influence trend of the fines content (*FC*). Some scholars [3,10,11,12] argue that *φ_ps_* shows a “first increase, then decrease” trend with the increase in *FC*: Salgado [10] conducted drained triaxial tests on Ottawa sand (rounded quartz sand) and found that, when the *FC* increased from 0% to 20%, fine particles filled the pores of coarse particles and enhanced the interlocking effect, leading to an increase in φ_ps_ from 30.1° to 33.0°; when the *FC* exceeded 20%, excess fine particles damaged the force-transferring skeleton of coarse particles, and *φ_ps_* began to decrease. Carraro [3] also confirmed in their study on quartz sand–non-plastic silt mixtures that, within the range of *SR* = 2~6, the peak value of *φ_ps_* always appeared at *FC* = 20%~30%, and, after the peak, *φ_ps_* decreased monotonically with the further increase in *FC*. Other scholars [13,14,15], however, hold that *φ_ps_* presents a “monotonically decreasing” trend with the *FC*: Li [14] carried out tests on South China Sea coral sand (easily crushable) and showed that, when the *FC* increased from 0% to 50%, fine particles aggravated the crushing of coral sand particles, resulting in a continuous decrease in *φ_ps_* from 39.96° to 26.60° without an “increasing stage”; Xiao [13,15] also found in their research on angular crushed glass sand that, when both sand and silt were angular, *φ_ps_* decreased by 3.2° as the *FC* increased from 0% to 15%, showing a consistent weakening trend.

Regarding the research on critical strength (*φ_cs_*), there is also a certain degree of controversy at present, which centers on whether the *FC* is related to *φ_cs_*. Some scholars [16,17,18,19] argue that *φ_cs_* is basically unrelated to the *FC*. Others [20,21], however, maintain that *φ_cs_* is closely related to the *FC*: in the coral sand tests by Li [20], as the *FC* increased from 0% to 50%, *φ_cs_* decreased from 32.9° to 25.6°; Xiao [21] found that, when the *FC* increased from 0% to 15%, *φ_cs_* also increased by 10%. The main reason for the aforementioned contradictions lies in the shape discrepancy between coarse and fines particles [19,21,22]: specifically, when the shape discrepancy between coarse and fine particles is small, the addition of fine particles with a similar shape does not alter the interlocking skeleton of the coarse particle structure itself, so *φ_cs_* does not change with the *FC*; when the shape discrepancy between coarse and fine particles is large, the incorporation of fine particles significantly alters the interlocking structure of the mixture—when subrounded angular fine particles are added to rounded coarse particles, the fine particles enhance the mechanical interlocking between coarse particles, thereby significantly increasing *φ_cs_*; on the contrary, when fine particles are added to angular coral sand, the fine particles mainly play a lubricating role, which, in turn, leads to a decrease in *φ_cs_*.

Meanwhile, it can be observed that the existing studies mostly focus on the influence of the *FC* on the strength of mixtures, while studies on the changes in mixture strength characteristics caused by variations in the *SR* remain scarce. As another core index regulating the strength of binary mixtures, the *SR* does not act independently on strength evolution; instead, it exhibits a significant coupling effect with the *FC* [11,19,23,24,25]. Essentially, by altering the size, distribution, and connectivity of coarse particle pores, the *SR* directly determines the “effective filling threshold” of fine particles: when the *SR* is low, a small *FC* is sufficient to fill the pores and optimize skeleton interlocking; when the *SR* is high, more fine particles are required to fill the skeleton. This coupling relationship implies that, if the *FC* is studied in isolation from the *SR*, it is easy to misjudge the “shift in strength trends caused by *SR* differences” as the inherent action law of the *FC* itself, thereby further exacerbating the contradictions in the macroscopic conclusions. Therefore, it is essential to systematically conduct mechanical tests on binary mixtures to investigate the joint influence of the *FC* and *SR*.

Most of the aforementioned studies summarize the strength and deformation characteristics of sand–fines mixtures based on the macroscopic phenomena observed in laboratory tests. However, macroscopic tests are essentially “black-box observations”—they can only capture macroscopic responses such as stress–strain and volume change, but fail to reveal the micro-interaction mechanisms underlying strength evolution. This has become a bottleneck in explaining the contradictions in the macroscopic conclusions and quantifying the coupling effect between the *FC* and *SR*. The discrete element method (*DEM*) [26] precisely compensates for this limitation. As a supplementary tool to macroscopic tests, it enables the intuitive observation of the evolution of particle contact types [27,28], force chain transmission [29,30], and fabric anisotropy [31] during shearing, thereby explaining the micro-nature of macroscopic phenomena. In recent years, some scholars have conducted research on sand–fines mixtures using the *DEM:* Zhu [19] quantified the proportion and force-bearing characteristics of coarse–coarse (cc), coarse–fine (cf), and fine–fine (ff) contacts under different *FC* and *SR* conditions through *3D-DEM* simulations, and clarified the correlation between the “unimodal trend of φₚ with *FC*” and the evolution of the contact network; Liu and Yan [32] proposed a strong contact identification method based on subnetwork division, and verified the dominant role of the strong contact system in the strength of sand–fines mixtures via the *DEM*; Cao [33] analyzed the coupling effect of the intermediate principal stress and *FC* on the microscopic force chains of binary mixtures using the *DEM*.

While numerous scholars have explored the mechanical properties of sand–fines mixtures via laboratory tests or *DEM* simulations, the existing studies still have notable limitations. On one hand, most works focus on single-factor analysis—either investigating the effect of the *FC* alone or the *SR* in isolation—without considering the combined and synergistic influence of the *FC* and *SR* on the macro–micro mechanical response of the mixtures. This narrow focus fails to capture the holistic regulatory mechanism of these two key factors, leaving a gap in our understanding of their coupled effects. On the other hand, studies relying solely on the *DEM* to explore microscopic mechanisms often suffer from a disconnect between the simulation and experiment: the selection of *DEM* models and core parameters typically depends on empirical methods, rather than being calibrated and verified against macroscopic test data. This lack of experimental validation reduces the reliability of microscopic mechanism interpretations, as the simulated results may deviate from the actual mechanical behavior of the material. These unresolved limitations underscore the need for a more integrated approach to address the research gaps.

Based on this, this study investigates the mechanical properties of sand–fines mixtures from both the macroscopic experimental and microscopic simulation perspectives. First, laboratory triaxial tests are conducted to study the strength indicators (peak friction angle *φ_ps_*, and critical-state friction angle *φ_cs_*) and deformation modes (dilatancy–contraction characteristics) of sand–fines mixtures under different fines contents (*FC* = 0.1, 0.2, 0.3, 0.5, and 0.7) and particle size ratios (*SR* = 9.1, 18.7, and 39.7). Then, from the micro-perspective, discrete element simulations are performed to observe the evolution of particle contact types (coarse–coarse, coarse–fine, and fine–fine) and the laws of force chain transmission, and to explore the mechanical mechanisms behind the mixture’s strength indicators. This systematic approach aims to clarify the regulatory laws of the fines content and particle size ratio on the mechanical properties of sand–fines mixtures, providing support for the optimization of sand–fines mixture proportions and the control of the anti-sliding stability in engineering practices.

## 2. Materials and Testing Program

### 2.1. Testing Materials

**Figure 1:**
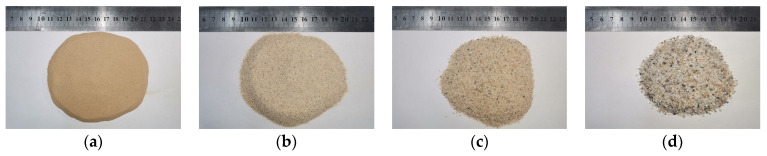
Images of coarse and fine particles: (**a**) Fine; (**b**) Sand-A; (**c**) Sand-B; and (**d**) Sand-C.

**Figure 2:**
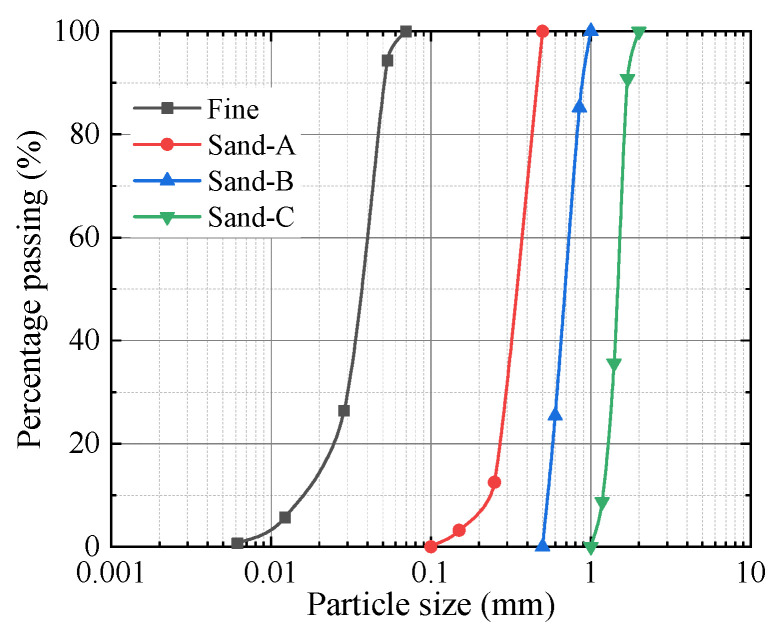
Grain size distributions of binary mixtures.

In this study, natural river sand was selected as the experimental material. Through sieving the river sand, three types of sandy soils with different particle sizes and one type of silty soil were obtained. The comparison of their particle sizes and particle size distributions is presented in Figure 1 and Figure 2, while the corresponding physical and mechanical parameters are listed in Table 1.

To investigate the effects of fines content and particle size ratio, the aforementioned silty soil was defined as “fine particles” and the three types of sandy soils as “coarse particles” in the experiment. Binary mixtures with three different particle size ratios were prepared by mixing the fine particles with any one type of coarse particles. For the binary mixtures with each particle size ratio, five gradients of fines content (i.e., 0.1, 0.2, 0.3, 0.5, and 0.7) were set, respectively. The physical and mechanical parameters of each mixture are shown in Table 2. Meanwhile, medium-dense sand and dense sand specimens with relative densities of 50% and 80%, respectively, were prepared to investigate the dilatancy characteristics of the sand–silt mixtures.

### 2.2. Testing Procedure

In this experiment, the Hollow Cylinder Torsion System manufactured by GDS Instruments (Hook, Hampshire, UK) was employed for testing. The axial displacement range of this system is significantly larger than that of conventional triaxial testing systems, which ensures that the sandy soil can be sheared to the critical state as much as possible. The specimens used in the experiment were cylindrical with a uniform specification of 50 mm in diameter and 100 mm in height, and were prepared by the Moist Tamping Method [34,35]. This method enables the uniform mixing of sand and silt, which can minimize the segregation between sand and silt particles and ensure the quality of the specimens. During specimen preparation, the dried sand and silt were weighed in proportion and thoroughly mixed with distilled water, accounting for 5% of the total mass. Subsequently, the mixture was compacted into 5 layers to the specified density. In the process of specimen preparation, an under-compaction rate of 3% was applied between adjacent layers to eliminate density differences caused by layer boundaries [36]. After compaction of each layer, the thickness of the layer was measured to ensure the overall height of the specimen (100 mm) met the design requirement, and the mass of each layer was verified to avoid segregation of sand and silt particles. After the specimen preparation was completed, saturation was achieved first by flushing with carbon dioxide gas and then with deaired water. Backpressure saturation was performed by applying confining pressure and backpressure in stages, during which the effective confining pressure was maintained constant at 20 kPa. After the backpressure reached 200 kPa, the confining pressure and backpressure were kept stable to help dissolve the residual gas in the water until the degree of saturation exceeded 0.98, at which point saturation was considered complete. Finally, isotropic consolidation was conducted by applying an effective confining pressure of 100 kPa, and the consolidation process was deemed complete when the volume of the specimen became stable and no longer changed. After consolidation, the specimens were sheared at a constant vertical displacement rate of 0.1 mm per minute until the axial strain reached 40%. All specimens were maintained under drained conditions during the shearing process. Furthermore, no particle breakage was observed throughout all the tests in this study, indicating that the test conditions did not affect the integrity of the sand and silt particles.

## 3. Testing Results

### 3.1. Stress–Strain and Volumetric Change Responses

**Figure 3:**
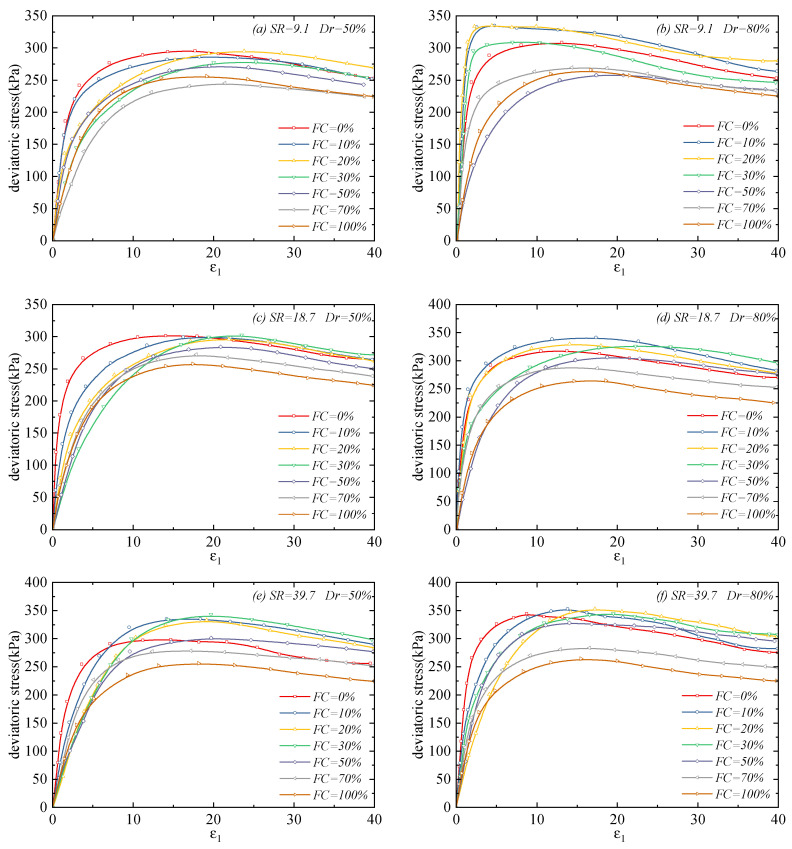
Stress–strain relationship of sand–silt mixtures under different fines contents and particle size ratios: (**a**) $SR=9.1\;D_{r}=50\%$; (**b**) $SR=9.1\;D_{r}=80\%$; (**c**) $SR=18.7\;D_{r}=50\%$; (**d**) $SR=18.7\;D_{r}=80\%$; (**e**) $SR=39.7\;D_{r}=50\%$; and (**f**) $SR=39.7\;D_{r}=80\%$.

Figure 3 presents the stress–strain relationships of sand–silt mixtures under different fines contents and particle size ratios. It can be seen from the figure that the shear stress of each specimen develops rapidly when the axial strain is small, until the deviatoric stress reaches the peak value. After the peak, the stress of the specimen decreases slightly and tends to a stable value, indicating that all binary mixtures exhibit strain-softening behavior under both medium-dense and dense conditions and reach the critical state.

Meanwhile, it can be observed that both the peak stress and softening degree of the binary mixtures are affected by the fines content and particle size ratio: when the *FC* is low, the peak strength increases with the increase in *FC*, and the stress-softening phenomenon becomes more obvious; when the *FC* increases to a certain value, the peak strength decreases with the further increase in fines content, and the stress-softening phenomenon is relatively weak. Additionally, at a low particle size ratio, the incorporation of a small number of fine particles leads to a sharp increase in peak strength and causes a more significant post-peak stress-softening behavior. However, for binary mixtures with a high particle size ratio, the change in fines content has a minor effect on their peak stress, and the softening phenomenon is not obvious simultaneously.

**Figure 4:**
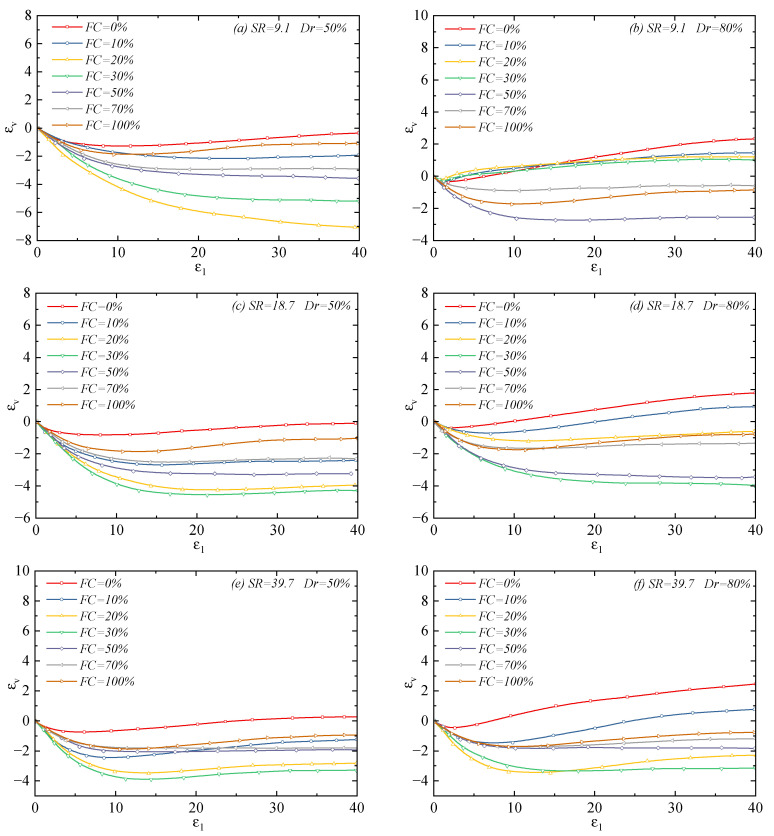
Volumetric strain–axial strain relationship of sand–silt mixtures under different fines content and particle size ratios: (**a**) $SR=9.1\;D_{r}=50\%$; (**b**) $SR=9.1\;D_{r}=80\%$; (**c**) $SR=18.7\;D_{r}=50\%$; (**d**) $SR=18.7\;D_{r}=80\%$; (**e**) $SR=39.7\;D_{r}=50\%$; and (**f**) $SR=39.7\;D_{r}=80\%$.

Figure 4 shows the volumetric strain evolution curves of the mixtures under a different *FC* and *SR*. It can be found that, similar to the effect on the peak strength, the *FC* also affects the volumetric strain evolution rule of the sand–silt mixtures. When the *FC* is low, with the increase in *FC*, the dilatancy of the mixture decreases, and the corresponding contraction becomes more obvious; when the *FC* exceeds a specific value, the dilatancy of the mixture becomes more obvious with the further increase in *FC*. This specific fines content is referred to as the threshold fines content (*FC_th_*) [4]. That is to say, when the *FC* is lower than the threshold fines content, the *FC* has an inhibitory effect on dilatancy; when the *FC* is higher than the threshold fines content, the *FC* has an enhancing effect on dilatancy. In this study, the method proposed by Rahaman [37] was used to calculate *FC_th_* with different particle size ratios:

(1) $$FC_{th}=0.4\left(\frac{1}{1+e^{0.5-0.13\chi }}+\frac{1}{\chi }\right)$$

Among them, $\chi =D_{10}/d_{50}$, $D_{10}$, and $d_{50}$ represent the effective particle size of coarse particles and the average particle size of fine particles, respectively. For the three sand–silt mixtures, the computed threshold fines contents are 22.0%, 30.7%, and 37.5%, respectively. This critical index is essentially consistent with the transitional fines content proposed by Cabalar [38], both representing the transition of the mixture from ‘coarse-grain skeleton-dominated’ to ‘fine-grain matrix-dominated. Therefore, for the mixture with *SR* = 18.7, when the *FC* is less than 30%, increasing the *FC* makes the mixture contraction more obvious; when the *FC* is more than 30%, increasing the *FC* causes more significant dilatancy, as shown in Figure 4.

Furthermore, experimental observations indicate that, compared with pure silt (*FC* = 100%), the dilatancy characteristics of the three types of pure coarse sands (*FC* = 0%) are significantly more prominent. This difference arises from the distinct disparities in the frictional properties and anti-sliding capacity between coarse and fine particles: the three types of coarse particles used in this study are all angular natural river sand with rough surfaces and irregular shapes. These particles tend to form interlocking structures between grains, which require overcoming greater interparticle resistance during shearing, making relative slippage difficult to occur. Therefore, they tend to release shear stress through volumetric expansion. In contrast, pure fine particles exhibit relatively weak interlocking effects, allowing easy relative slippage during shearing without requiring significant volumetric expansion to complete the stress transfer, manifesting as shear contraction or weak shear dilation characteristics.

### 3.2. Stress–Dilatancy Relationship

**Figure 5:**
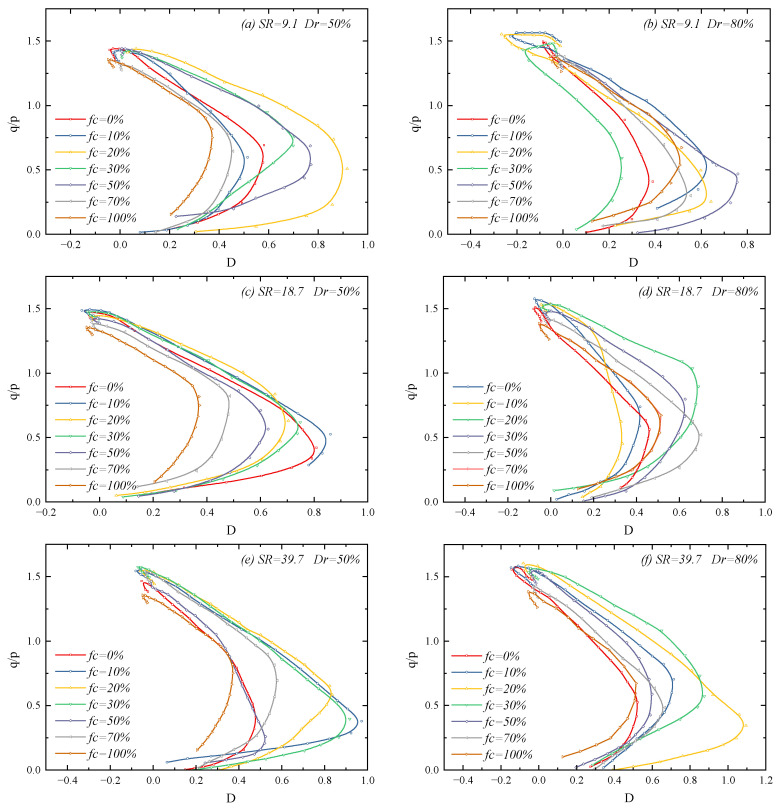
Stress–dilatancy relationship of sand–silt mixtures under different fines content and particle size ratios: (**a**) $SR=9.1\;D_{r}=50\%$; (**b**) $SR=9.1\;D_{r}=80\%$; (**c**) $SR=18.7\;D_{r}=50\%$; (**d**) $SR=18.7\;D_{r}=80\%$; (**e**) $SR=39.7\;D_{r}=50\%$; and (**f**) $SR=39.7\;D_{r}=80\%$.

Figure 5 presents the stress–dilatancy relationship of mixtures under different fines content and particle size ratios. It can be observed that all mixtures exhibit contraction behavior (*D* > 0) at the initial stage of dilatancy development; subsequently, the contraction phenomenon gradually diminishes. When reaching the phase transformation point, the specimens begin to exhibit dilatancy characteristics. Additionally, it can be noted that, before the threshold fines content, an increase in *FC* leads to a more obvious contraction of the specimens before the phase transformation point; after the threshold fines content, an increase in *FC* content results in less contraction of the mixtures before the phase transformation point. This phenomenon indicates that the *FC* not only affects the magnitude of dilatancy of the mixtures after the phase transformation point (as shown in Figure 4) but also influences the contraction phenomenon of the mixtures before the phase transformation point.

In addition, it is observed that, after the specimens reach the peak dilatancy point, their stress–dilatancy curves change and exhibit a “hook” shape when approaching the critical state. Furthermore, the hooks of most specimens in Figure 5 curve downward, meaning that the stress degradation rate is greater than the dilatancy ratio degradation rate. However, it can be seen from Figure 5b that, when the particle size ratio is 9.1 and the relative density is 80%, the “hooks” curve upward for specimens with a fines content of 0–30%; in this case, the dilatancy ratio degradation rate is greater than the stress ratio degradation rate. The reason for this phenomenon is that the peak dilatancy ratio of specimens with upward-curving “hooks” is significantly higher than that of other specimens. When these specimens reach the peak state, inter-particle sliding becomes more obvious, leading to a rapid degradation of the dilatancy ratio.

### 3.3. Peak State Friction Angle

For granular materials, the peak friction angle *φ_ps_* is defined as follows [39]:

(2) $$\varphi_{ps}=\frac{{\left({\sigma^{\prime }}_{1}/{\sigma^{\prime }}_{3}\right)}_{ps}-1}{{\left({\sigma^{\prime }}_{1}/{\sigma^{\prime }}_{3}\right)}_{ps}+1}$$

**Figure 6:**
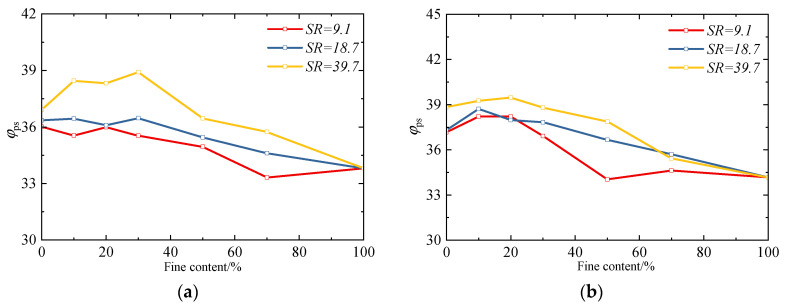
Effect of fines content and particle size ratios on the peak state friction angle: (**a**) *D_r_* = 50%; and (**b**) *D_r_* = 80%.

Among them, ${\sigma^{\prime }}_{1},{\sigma^{\prime }}_{3}$ represent the maximum principal stress and minimum principal stress (both in kilopascal, kPa), respectively. Figure 6 presents the variation trend of the peak friction angle of sand–silt mixtures with fines content. It can be observed that the specimens with a relative density of 80% exhibit a higher peak stress, indicating that sand–silt mixtures with a higher relative density possess a more robust “skeletal structure”. During shearing, a greater inter-particle interlocking resistance needs to be overcome. In contrast, sandy soils with a lower relative density have a loose particle arrangement; their skeletal structure is prone to deformation, and the peak stress required for relative sliding between particles is lower.

Meanwhile, it can be observed that, regardless of the particle size ratio, the sand–silt mixtures all exhibit a trend where the peak internal friction angle first increases and then decreases with the increase in fines content, which is consistent with the *DEM* simulation results by Zhu [19]. When the fines content is low, fine particles fill the pores between coarse particles, reducing the “contact point defects” among coarse particles and further forming a more complete force chain network. As a result, the shear stress can be more uniformly transmitted through the coarse particle skeleton during the shearing process, avoiding strength loss caused by local contact damage and thereby enhancing the friction angle corresponding to the peak stress.

In addition, it can be observed that the peak stress of mixtures with a high *FC* (e.g., *FC* = 50%, 70%, and 100%) is significantly lower than that of pure coarse particles. The reason for this phenomenon lies in the fact that, compared with the angular coarse particles used in this study, the fine particles have smoother surfaces and a stronger “lubricity” between particles, making relative sliding more likely to occur during the shearing process, which, in turn, leads to a substantial reduction in the overall shear resistance. When the fines content increases to 50% or above, the proportion of fine particles in the mixture increases significantly, and the particle contact type transitions from “coarse–coarse contact dominance” to “fine–fine contact and coarse–fine contact dominance”. The smooth surfaces of fine particles result in a lack of effective interlocking in such contacts, and the frictional resistance between particles is much lower than that between coarse particles. Ultimately, this leads to the peak stress of mixtures with a high fines content being significantly lower than that of pure coarse particles.

### 3.4. Critical-State Friction Angle

With continuous shearing, the volumetric strain and stress ratio remain constant, and the specimen reaches the critical state. The critical-state friction angle *φ_cs_* of granular materials can be calculated by the following formula:

(3) $$\varphi_{cs}=\frac{{\left({\sigma^{\prime }}_{1}/{\sigma^{\prime }}_{3}\right)}_{cs}-1}{{\left({\sigma^{\prime }}_{1}/{\sigma^{\prime }}_{3}\right)}_{cs}+1}$$

It can be observed from Figure 4 that the stress–strain curves of different specimens have all stabilized when the axial strain reaches 40%. Therefore, in this study, the state corresponding to an axial strain of 40% is taken as the critical stress state.

**Figure 7:**
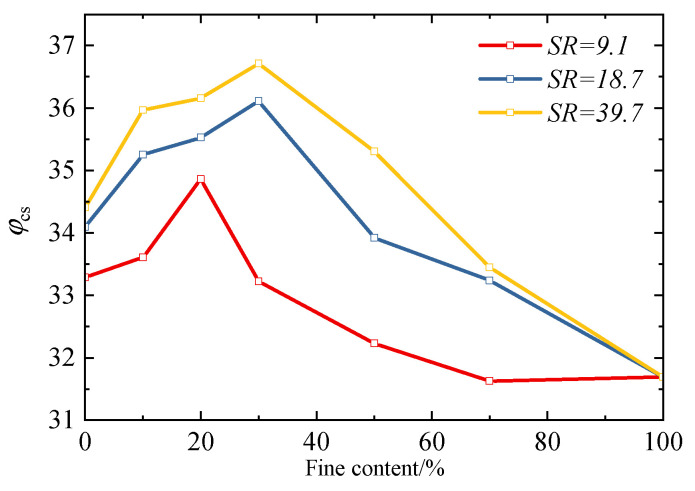
Effect of fines content and particle size ratios on the critical-state friction angle.

Figure 7 presents the evolution trend of the critical-state friction angle *φ_cs_* for specimens with different fines content and particle size ratios. It can be observed that both the fines content and particle size ratio exert a significant influence on the *φ_cs_* of the specimens. Overall, with the increase in fines content, *φ_cs_* exhibits a trend of first increasing and then decreasing, which differs slightly from the results by Li [20] (*φ_cs_* decreases gradually with the increase in fines content, showing a negative exponential relationship) and Yilmaz [4] (*φ_cs_* remains basically constant at a low and high fines content). This discrepancy arises from the particle morphological characteristics of the test materials: the coarse particles used in this study are angular natural river sands, featuring rough surfaces and strong interlocking capabilities. The incorporation of fine particles does not merely “fill pores” or “destroy the skeleton”: when the *FC* is low, fine particles fill the contact defects between coarse particles, enhancing the interlocking effect among coarse particles and thus increasing *φ_cs_* with the rise in *FC*; when the *FC* exceeds the threshold, excessive fine particles isolate the coarse particles, weakening the force transmission capacity of the coarse particle skeleton. Meanwhile, the interlocking effect of the *FC*s themselves is relatively weak, leading to a decrease in *φ_cs_* as the *FC* increases. This “first increase, then decrease” trend is essentially the result of the dynamic competition between two effects in the angular particle system: “fine particle filling reinforcement” and “skeleton isolation weakening”. It further confirms that the skeleton-filling effect of fine particles is the key mechanism regulating the critical state of binary mixtures.

In addition, it can be observed that the variation laws of the critical-state friction angle for specimens with different particle size ratios also exhibit slight differences. For the specimens with *SR* = 9.1, the critical-state friction angle reaches its peak when the fines content is 20%, followed by a subsequent decrease. In contrast, for the specimens with particle size ratios of 18.7 and 39.7, their critical-state friction angles reach the peak when the fines content is 30%. This phenomenon is attributed to the fact that binary mixtures with different *SR*s correspond to distinct *FC_th_* values. Specifically, the *FC_th_* values for mixtures with *SR* = 9.1, 18.7, and 39.7 are 22.0%, 30.7%, and 37.5%, respectively. Therefore, for mixtures with *SR* = 9.1, when *FC* = 20% (close to its *FC_th_* of 22.0%), their critical state is nearly reaching the threshold; for mixtures with *SR* = 18.7 and 39.7, *FC* = 30% is close to their respective *FC_th_* (30.7% for *SR* = 18.7, and 37.5% for *SR* = 39.7), so their critical states also approach the threshold at this *FC*.

Meanwhile, it can be observed that the particle size ratio also affects the transitional fines content range of sand–silt mixtures. Yilmaz’s research [4] results indicate that the critical-state friction angle of sand–silt mixtures does not change with the *FC* when the *FC* is low or high; however, when the *FC* falls within a specific range, the critical-state friction angle of the mixture changes linearly with the *FC*. This range is referred to as the transitional fines content range. The transitional ranges of materials with different particle size ratios also differ. Affected by the particle shape and mineral composition, although the variation law of the critical-state friction angle in the test results of this study is slightly different from that of Yilmaz’s research [4], it can be seen from Figure 7 that, similar to Yilmaz’s findings, the larger the particle size ratio is, the wider the transitional range is. This is mainly reflected in the following aspect: at a relatively high fines content, the critical-state friction angles with a particle size ratio of 9.1 are relatively close when the *FC* is 70% and 100%, indicating that the influence of coarse particles on the mixture is already minimal at this point. At this point, its transitional fines content range is approximately from 30% to 60%. However, for mixtures with larger particle size ratios (e.g., 18.7 and 39.1), the critical-state friction angle of the mixture with a 70% fines content is still higher than that of the mixture with a 100% fines content, which suggests that the coarse particles still play a certain reinforcement role at this stage [40]. This indicates that materials with a larger particle size ratio have a wider transition range, which is approximately 30% to 70%.

## 4. DEM Modeling

To further gain an in-depth understanding of the influence of the fines content (*FC*) on the mechanical behaviors of sand–fines mixtures from the meso–micro perspective, this section employs the discrete element method (*DEM*) to conduct a simulation and analysis on the mechanical behaviors of the aforementioned binary mixtures.

### 4.1. Contact Model

**Figure 8:**
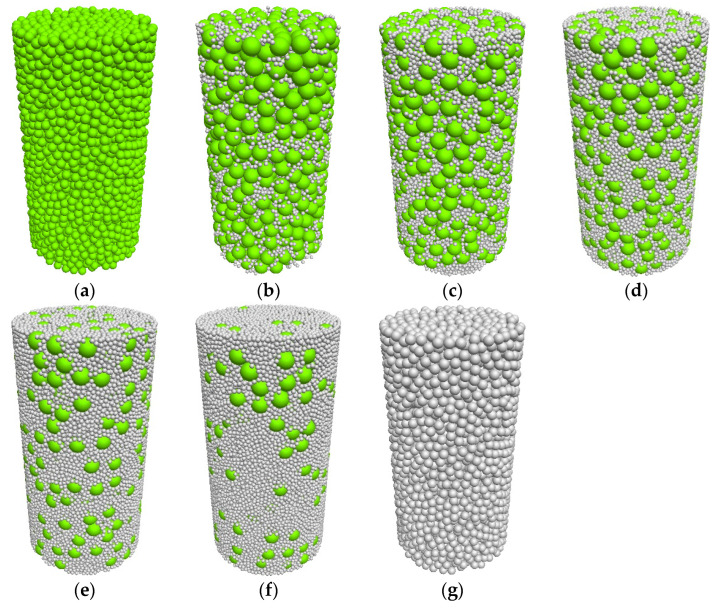
Typical numerical sample [45]: (**a**) *FC* = 0%; (**b**) *FC* = 10%; (**c**) *FC* = 20%; (**d**) *FC* = 30%; (**e**) *FC* = 50%; (**f**) *FC* = 70%; and (**g**) *FC* = 100%.

This section aims to analyze the influence law of different fines contents (*FC*s) on the mechanical behaviors of the mixtures from a meso–micro perspective. Meanwhile, to balance the computational accuracy and efficiency, the particle size ratio (*SR*) in this section is set to 4, meaning the radius of coarse particles is 0.1 mm and that of fine particles is 0.025 mm. The linear contact model is adopted for inter-particle contacts, and numerous studies [41,42] have verified that this model can accurately reproduce the mechanical properties of granular materials. The normal contact stiffness is calculated using the formula *k_n_ = k*_0_*r*, where *k*_0_ is the stiffness constant. The values of *k*_0_ are set to 1.0 × 10^3^ MPa for coarse particles and 4.0 × 10^3^ MPa for fine particles, with the specific parameters detailed in Table 3, consistent with the literature [32]. Similarly, to ensure that mixture specimens with different fines contents have the same relative density, a specimen preparation method that controls the particle friction coefficient is adopted [43,44]. During the isotropic consolidation process, the particle friction coefficient is set to 0, thereby obtaining the densest specimens (only this one density is simulated in this section due to space limitations). The consolidated specimens are shown in Figure 8.

**Figure 9:**
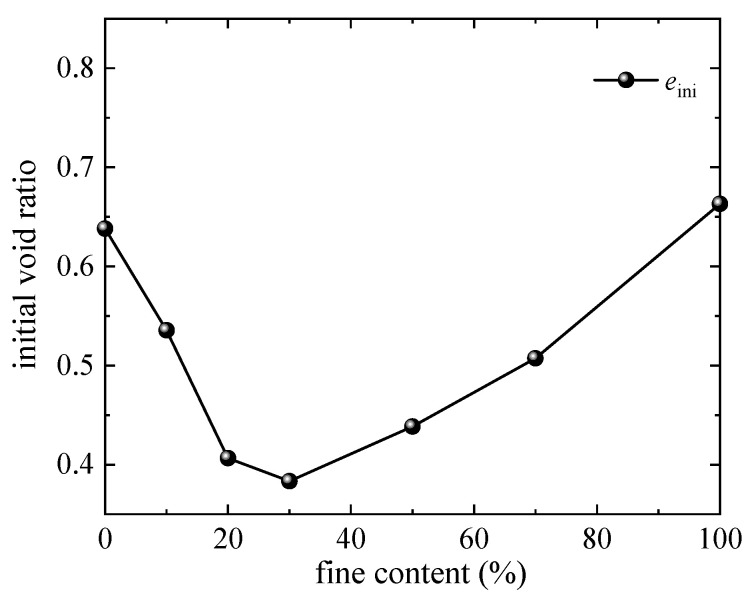
Relationship between initial void ratio and fines content.

Figure 9 presents the curve of the initial void ratio of sand–silt mixtures varying with the *FC*. The initial void ratio exhibits a trend of first decreasing and then increasing with the *FC*: it reaches the minimum value when the *FC* increases to approximately 30%, and gradually rises as the *FC* further increases. This is consistent with both the experimental results [46,47] and existing simulation results, indicating the effectiveness of the established model.

Before shearing, the particle–particle friction coefficient is uniformly set to 0.5, while the wall–particle friction coefficient is kept at 0. The upper and lower walls move toward each other at a rate of 0.005 m/s for shear loading, and a servo-control mechanism is adopted to maintain a constant confining pressure. To ensure that the sand–silt mixture remains in a quasi-static state throughout the shearing process, the shear rate must satisfy the requirement [48] of the inertia parameter $I=\varepsilon_{1}^{\prime }d\sqrt{\rho /p}<2.5\times 10^{-3}$, where *I* is jointly determined by the axial strain rate $\varepsilon_{1}^{\prime }$, particle diameter *d*, and particle density *ρ* (see Table 3 for the values of d and *ρ*). The calculation results show that *I* < 2.5 × 10^−3^; thus, the specimen maintains a quasi-static equilibrium state during the entire shearing process. In addition, to ensure that the specimens reach the critical state, all specimens are sheared until the axial strain reaches 40%.

### 4.2. Analysis of Micro-Mechanical Responses

**Figure 10:**
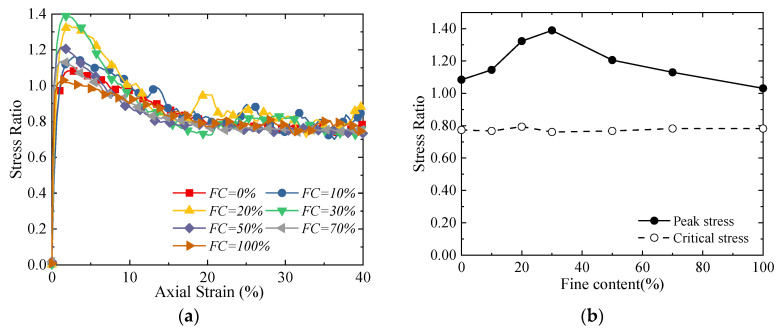
Evolution of stress–strain relationship with respect to *FC* [32]: (**a**) stress–strain relationship from *DEM* simulation; and (**b**) peak and critical-state stress ratios.

Figure 10 presents the diagram illustrating the influence of different *FC*s on the macro stress–strain relationship of binary mixtures. As can be seen from Figure 10a, with the increase in axial strain, the stress ratio of all specimens rises rapidly in the early stage of shearing, then gradually decreases after reaching the peak value and tends to stabilize—all specimens exhibit obvious strain-softening behaviors. The discrete element method (*DEM*) simulation results are highly consistent with Zhu’s calculation results [19] and are also close to the experimental results in the previous section. When the fines content increases from 0% to 30% (Figure 10b), the peak stress ratio increases gradually, indicating a gradual enhancement in the shear strength of the specimens; when the *FC* further increases from 30% to 100%, the peak stress ratio decreases gradually, which means the peak strength reduces progressively. In addition, it can be observed that the critical-state stress ratio is insensitive to changes in the fines content, which indicates that the specimens will eventually reach the same critical state regardless of the variations in the fines content. This phenomenon is inconsistent with the macro experimental results, and the reason lies in that both the coarse and fine particles used in the simulation are spherical, with no difference in shape between them. This further proves that the particle shape is a key variable affecting the critical state [49].

The coordination number refers to the number of contacts between a particle and its surrounding particles, while the average coordination number reflects the tightness of inter-particle contacts. As a fundamental parameter for characterizing the microstructural characteristics of sand–silt mixtures, it can be expressed as follows [50]:

(4) $$C_{m}=\frac{2N_{c}-N_{1}}{N_{p}-N_{0}-N_{1}}$$

where *N_c_* and *N_p_* represent the total number of contacts and the total number of particles, respectively; and *N*_0_ and *N*_1_ denote the number of particles with no contacts and only one contact, respectively. Particles with no contacts or only one contact cannot effectively form a stable force transmission network with other particles. Therefore, they do not participate in bearing external loads or transmitting internal forces. To more accurately reflect the actual force-bearing state of sand–silt mixtures, these particles are excluded when calculating the coordination number.

**Figure 11:**
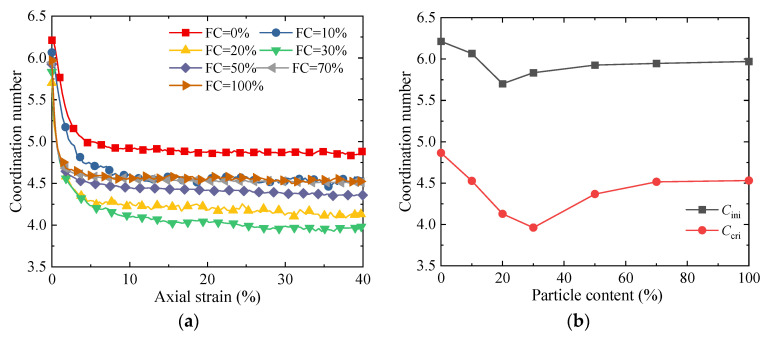
The evolution of the mechanical coordination number with respect to *FC* [32]: (**a**) relationship between coordination number and axial strain; and (**b**) coordination number in initial state and critical state.

Figure 11 presents the evolution of the coordination number. The results show that, for specimens with all fines contents, the coordination number gradually decreases as shearing proceeds and tends to stabilize after reaching the critical state. When *FC* < 30%, the coordination number decreases as the fines content increases; however, when *FC* > 30%, the coordination number increases with the increase in fines content—this is contrary to the common understanding. Generally, the void ratio is negatively correlated with the coordination number—i.e., the larger the void ratio, the smaller the coordination number [9,51]. The reason for this “abnormal phenomenon” is as follows: under the condition of a low fines content (when *FC* < 30%), a large number of fine particles are located in the pores formed by coarse particles, resulting in fewer contacts between these fine particles and the surrounding particles. Therefore, under the condition of a low fines content, as the fines content increases, the skeleton formed by coarse particles becomes loose; meanwhile, the number of particles with no contacts or only one contact increases significantly. These two factors jointly lead to a decrease in the coordination number.

Inside the sand–silt mixture, particles form a highly inhomogeneous force transmission network through complex inter-particle contact interactions. During the shearing process, the contact states between the particles and the force transmission network continuously adjust and evolve to adapt to the changes in the external loads. The fines content plays a crucial regulatory role in this evolutionary process, and analyzing the evolution law of the contact force network is conducive to gaining a deeper understanding of the strength source of binary mixtures. Different from the conventional method of classifying strong and weak contact networks (which uses the overall average contact force as the threshold), this study adopts the average contact force of each of the three contact sub-networks [32] as the threshold to classify strong and weak contact networks. This approach is based on the consideration that there are significant differences in contact forces between coarse–coarse (CC), coarse–fine (CF), and fine–fine (FF) contacts, and aims to comprehensively evaluate the contribution of different types of contacts to the mechanical behaviors of binary mixtures.

**Figure 12:**
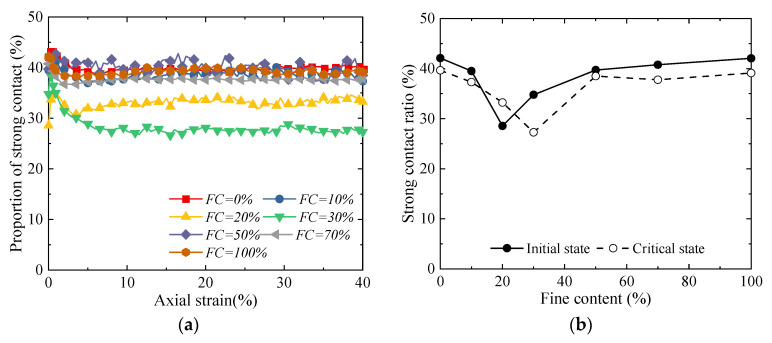
Evolution of proportion of strong contacts [32]: (**a**) relationship between strong contact ratio and axial strain; and (**b**) proportion of strong contacts in initial state and critical state.

**Figure 13:**
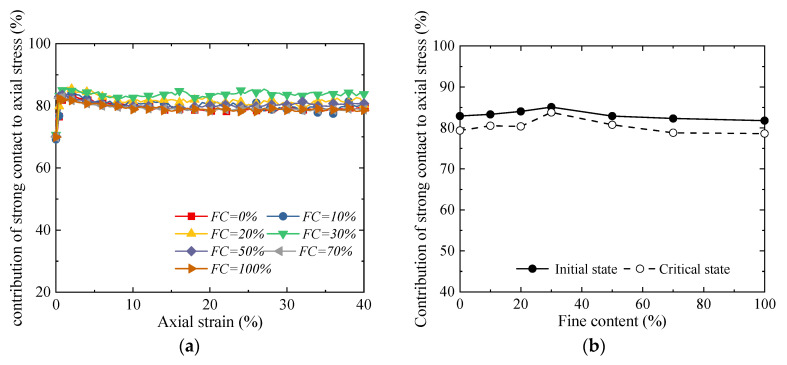
Contribution of strong contacts to axial stress [32]: (**a**) relationship between axial stress contribution of strong contacts and axial strain; and (**b**) relationship between the contribution of strong contacts to axial stress and fines content.

Figure 12 illustrates the variation law of the proportion of strong contacts with the fines content. Overall, the fines content exerts a significant regulatory effect on the structure of the particle contact network: as the fines content increases from 0% to approximately 30%, the proportion of strong contacts decreases continuously; when the fines content exceeds 30% and further increases to 100%, the proportion of strong contacts shows an upward trend (Figure 12). However, the law governing the contribution of strong contacts to the axial stress is exactly opposite to the variation in the proportion of strong contacts (Figure 13): at a fines content of around 30%, although the proportion of strong contacts is at its lowest level, their load-bearing contribution to the axial stress reaches the maximum. Meanwhile, the small number of strong contacts results in more weak contacts in the particle system. Although these weak contacts do not directly bear the main axial stress, they form a stable lateral support system, which effectively restrains the lateral deformation of the strong contact skeleton, thereby enhancing the overall shear strength and stability. Therefore, when the *FC* is 30%, a structure in which the “high-efficiency load-bearing” strong contact network and the “stable supporting” weak contact network cooperate with each other is formed inside the material—and this is the primary reason for its macro strength reaching the peak value.

Studies [52,53] have shown that the stress evolution of granular materials is closely related to the fabric tensor, and many scholars have established various fabric–stress relationships. Some scholars [54] argue that the strength evolution of granular materials exhibits a linear relationship with the anisotropy coefficient of the strong contact fabric. Using the aforementioned sub-network-based method for classifying strong contacts, the anisotropy coefficient of the strong contact fabric ($a_{c}^{s}$) can be expressed as follows:

(5) $$a_{c}^{s}=\sum_{m}W^{s,m}a_{c}^{s,m},\;a_{c}^{s,m}=\sqrt{\frac{3}{2}a_{ij}^{s,m}a_{ij}^{s,m}},\;a_{ij}^{s,m}=15/2\Phi_{ij}^{s,m\prime }$$

where *m* denotes the three contact sub-networks, namely, coarse–coarse (CC), coarse–fine (CF), and fine–fine (FF); $\Phi_{ij}^{s,m\prime }$ represents the deviatoric tensor of $\Phi_{ij}^{s,m}=\frac{1}{N^{s,m}}\sum_{c=1}^{N^{s,m}}n_{i}^{s,m}n_{j}^{s,m}$, where the superscript *s* specifically indicates “strong contacts”; $n_{j}^{s,m}$ stands for the branch vector of strong contacts within the “*m*”-type contact sub-network; $N^{s,m}$ refers to the number of strong contacts in the “*m*”-type contact sub-network; and $W^{s,m}$ denotes the weight coefficient corresponding to the “*m*”-type contact sub-network ($W^{s,m}=N^{s,m}/N^{s}$).

**Figure 14:**
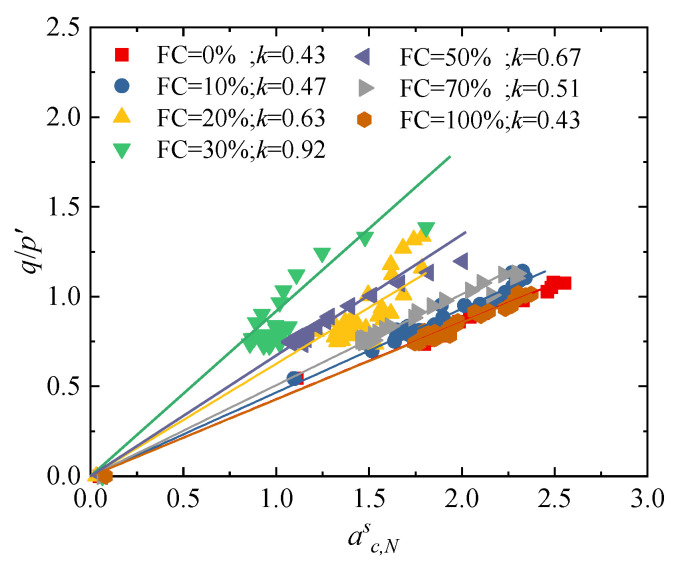
Stress–strong contact fabric relationship of specimens with different fines content.

Figure 14 presents the relationship between the deviatoric stress ratio and the anisotropy coefficient of the strong contact fabric for specimens with a different fines content. It can be observed that there is a linear relationship between the anisotropy of the strong contact fabric and stress ratio; as the fines content increases, the slope of the strong contact fabric–stress relationship first increases and then decreases. When the fines content is 30%, the anisotropy of the strong contact fabric reaches its minimum, and this minimum anisotropy corresponds to the highest strength of the specimen. This phenomenon indicates that, under this fines content, the weak contacts enhance the stability of the strong contacts in the form of “caging” or lateral confinement, thereby suppressing the instability of force chains and the rolling of particles.

Equation (5) only considers the relationship between the contact normal fabric anisotropy and strength. However, existing studies [28,32] have shown that both the mean normal contact force anisotropy and branch vector anisotropy also contribute to the strength of granular materials. This contribution is particularly significant for heterogeneous materials such as binary mixtures. To further account for their effects, the weight coefficient in Equation (5) is modified as follows:

(6) $$W^{s,m}=\frac{N^{s,m}{\bar{f}}_{n}^{s,m}{\bar{b}}^{s,m}}{N^{s}{\bar{f}}_{n}^{s}{\bar{b}}^{s}}/\sum_{m}\frac{N^{s,m}{\bar{f}}_{n}^{s,m}{\bar{b}}^{s,m}}{N^{s}{\bar{f}}_{n}^{s}{\bar{b}}^{s}}$$

where ${\bar{f}}_{n}^{s,m}$ denotes the average value of the strong contact forces in the “*m*”-type sub-network, while ${\bar{f}}_{n}^{s}$ represents the average value of the overall strong contact forces of the specimen, both in newton (N); and ${\bar{b}}^{s,m}$ stands for the average value of the branch vectors of the strong contacts in the “*m*”-type sub-network, and ${\bar{b}}^{s}$ refers to the average value of the branch vectors of the overall strong contacts of the specimen.

**Figure 15:**
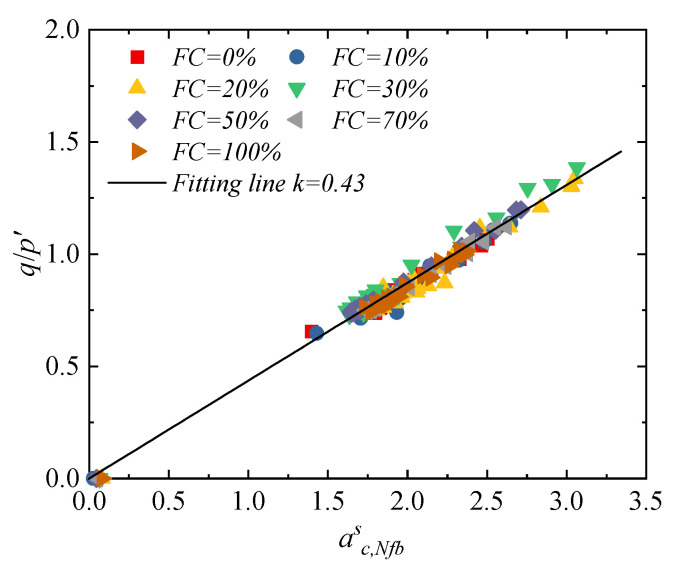
Stress-modified strong contact fabric relationship of specimens with different fines content.

By substituting Equation (6) into Equation (5), the modified relationship between the anisotropy parameter of the strong contact fabric and the stress ratio can be obtained, as shown in Figure 15. The results indicate that, under different fines content conditions, there exists a unique and significant linear correlation between the anisotropy of the strong contact fabric and the stress ratio, with a Pearson correlation coefficient [55] as high as 0.95. When describing the macro-mechanical response of binary mixtures, it is crucial to consider both the contact force anisotropy and branch vector anisotropy for establishing a unified fabric–stress relationship. Although the fines content significantly affects the macro-mechanical properties of granular materials, at the microscale, the strong contact network plays a similar core role in sand–silt mixtures with different fines contents. This finding not only reveals the unified mechanical mechanism of strong contacts in sand–silt mixtures but also provides a more unified and accurate theoretical framework and evaluation criterion for further exploring the micro–meso essence of the instability and failure of sand–silt mixtures.

## 5. Conclusions

The study focuses on sand–silt mixtures. By combining laboratory triaxial tests with discrete element method (*DEM*) simulations, it systematically investigates the effects of the fines content (*FC* = 0%, 10%, 20%, 30%, 50%, 70%, and 100%) and size ratio (*SR* = 9.1, 18.7, and 39.7) on the macroscopic mechanical properties and microscopic interaction mechanisms of the mixtures, and clarifies the correlation laws between macro- and microscopic parameters. The main conclusions are as follows:

(1) Both the peak friction angle (*φ_ps_*) and critical-state friction angle (*φ_cs_*) of sand–silt mixtures exhibit a trend of “first increase, and then decrease” with an increasing fines content. The peak inflection point of their variation curves corresponds to the threshold fines content. When the *FC* is below the threshold, fines fill the pores of coarse particles and enhance inter-particle interlocking, thereby improving the strength; when the *FC* exceeds the threshold, excessive fines disrupt the force-transmitting skeleton of coarse particles, leading to strength attenuation. Moreover, the smaller the *SR*, the lower the threshold *FC*, and the narrower the transitional range of the *FC* on the strength. Specifically, the threshold *FC* of mixtures with *SR* = 9.1 is approximately 10% lower than that of mixtures with *SR* = 18.1 and 39.7, and its transitional range of the *FC* is also approximately 10% narrower than theirs.
(2) The fines contents and size ratio jointly regulate the deformation mode of the mixtures: when the *FC* is below the threshold, the contraction behaviors of the mixtures becomes more significant with an increasing *FC*, while dilatancy is inhibited; when the *FC* is above the threshold, dilatancy gradually strengthens with an increasing *FC*. Additionally, mixtures with a smaller *SR* show more significant post-peak stress softening and greater dilatancy in the low *FC* range: Mixtures with *SR* = 9.1 exhibit more significant post-peak stress softening with a softening amplitude of 15–20% and greater dilatancy with a dilatancy ratio of 0.8–1.2; in contrast, mixtures with *SR* = 39.7 display weaker post-peak stress softening with a softening amplitude of only 5–8% and smaller dilatancy with a dilatancy ratio of 0.2–0.5.
(3) The results from microscopic simulations indicate that, when the fines content is approximately 30%, although the proportion of strong contacts is the lowest, their contribution to the axial stress is the highest. Meanwhile, a large number of weak contacts form a stable lateral support, effectively constraining the deformation of the strong contact skeleton. At this point, the coordination between load-bearing and support within the material is achieved, resulting in the peak macroscopic strength.
(4) By considering the influence of the contact number, average normal contact force, and average branch vector length on the weight coefficient of fabric anisotropy, a unique linear relationship between the fabric anisotropy of strong contacts and the stress ratio is established. This indicates that, at the microscopic scale, the strong contact network plays a similar core role in sand–silt mixtures with different fines contents, providing a unified theoretical framework and evaluation criterion for exploring the microscopic essence of instability and failure in sand–silt mixtures.

It should be noted that this study has certain limitations: although the macro-mechanical responses and micro-action mechanisms of sand–fines mixtures are explored through macro triaxial tests and *DEM* simulations, the influence of the particle shape is not considered in the *DEM* simulation process. Specifically, the current simulation adopts uniformly spherical particles, lacking exploration on the impact of the shape differences between coarse and fine particles (e.g., angular coarse particles vs. irregular fine particles) on the mechanical mechanisms of sand–fines mixtures.

In terms of future research directions, more *DEM* simulations of coarse and fine particles with different shapes can be carried out—for example, introducing angularity parameters for coarse particles and irregular shape models for fine particles—to more intuitively explore how the particle shape modulates the contact network evolution, force chain transmission, and, ultimately, the macro-mechanical properties of sand–fines mixtures. This will help further improve the comprehensiveness and practicality of the research on the mechanical behavior of sand–fines mixtures.

## Figures and Tables

**Table 1:** Specific weight of particle.

Soils	Sand-A	Sand-B	Sand-C	Fine
Median particle diameter *D*_50_/mm	0.338	0.693	1.46	0.037
Specific gravity *G_S_*/g·cm^−3^	2.654	2.642	2.640	2.653

**Table 2:** Void ratios of binary mixtures.

SR *	FC */%	e_max_	e_min_	$D_{r}$/%	e_0_	SR	FC/%	e_max_	e_min_	$D_{r}$/%	e_0_
9.1	0	1.235	0.779	50	1.007	18.7	0	1.152	0.746	50	0.949
				80	0.870					80	0.827
	10	1.130	0.669	50	0.899		10	1.013	0.603	50	0.808
				80	0.761					80	0.685
	20	1.089	0.568	50	0.829		20	0.853	0.498	50	0.676
				80	0.672					80	0.569
	30	1.016	0.534	50	0.775		30	0.881	0.458	50	0.669
				80	0.630					80	0.542
	50	0.937	0.449	50	0.693		50	0.848	0.468	50	0.658
				80	0.546					80	0.544
	70	1.080	0.560	50	0.820		70	0.908	0.546	50	0.727
				80	0.664					80	0.618
39.7	0	1.140	0.733	50	0.937	39.7	10	1.019	0.596	50	0.808
				80	0.815					80	0.681
	20	0.930	0.442	50	0.686		30	0.852	0.341	50	0.597
				80	0.540					80	0.443
	50	0.826	0.377	50	0.602		70	0.897	0.530	50	0.713
				80	0.467					80	0.603
	100	1.138	0.736	50	0.937		100	1.138	0.736	80	0.817

* *SR* refers to the particle size ratio, where *SR* = *D*_50_/*d*_50_; *FC* refers to the fines content; *e_max_* = the maximum void ratio; *e_min_* = the minimum void ratio; *D_r_* = the relative density (expressed as a percentage); and *e*_0_ = the initial void ratio.

**Table 3:** Micromechanical parameters.

Parameter	Value
particle radius *r*/mm	0.1, 0.025
particle density *ρ*/kg·m^−3^	2600
particle–particle friction coefficient *μ*	0, 0.5
Wall–particle friction coefficient *μ*_wp_	0
stiffness constant *k*_0_/MPa	1.0 × 10^3^, 4.0 × 10^3^

## Data Availability

The original contributions presented in this study are included in the article. Further inquiries can be directed to the corresponding author.
